# 

**Published:** 1891-06-27

**Authors:** 


					AND INVALIDS.
N<\ 24(8. Vol, X.] Satttbdat,
June 27th, 1891.
? [One Penot.

				

